# Congenital Family Steatorrhea

**Published:** 1914-01

**Authors:** 


CONGENITAL FAMILY STEATORRHEA. Garrod and Hurtley *Quarterly Journal of Medicine,* January, 1913. Boy, aged eight, subject from infancy to the passage from the bowel of liquid fat, which solidifies on cooling. He is the second of five children, whose parents are first cousins. The fifth child, now dead, was similarly affected. There are no other morbid manifestations and no indication of any disease. However much or little fat be taken, some 25 per cent. is thus lost in the stools, and it is supposed that he is the subject of a rare inborn error of fat absorption, "probably a Mendelian recessive characteristic," although so far investigation has not determined wherein the error lies.

